# Metabolomics Coupled with Pathway Analysis Provides Insights into Sarco-Osteoporosis Metabolic Alterations and Estrogen Therapeutic Effects in Mice

**DOI:** 10.3390/biom12010041

**Published:** 2021-12-28

**Authors:** Ziheng Wei, Fei Ge, Yanting Che, Si Wu, Xin Dong, Dianwen Song

**Affiliations:** 1 Department of Orthopedics, Shanghai General Hospital, School of Medicine, Shanghai Jiao Tong University, Shanghai 201620, China; wzh_smmu@163.com; 2 School of Medicine, Shanghai University, Shanghai 200444, China; gefeichem@163.com (F.G.); Cheyantingdei@163.com (Y.C.); 3 College of Sciences, Shanghai University, Shanghai 200444, China; 4 Institute of Translational Medicine, Shanghai University, Shanghai 200444, China; 5 Department of Genetics, Stanford University School of Medicine, Stanford, CA 94305, USA

**Keywords:** postmenopausal osteoporosis, sarcopenia, skeletal muscles, estrogen, metabolomics

## Abstract

Postmenopausal osteoporosis (PMOP) and sarcopenia are common diseases that predominantly affect postmenopausal women. In the occurrence and development of these two diseases, they are potentially pathologically connected with each other at various molecular levels. However, the application of metabolomics in sarco-osteoporosis and the metabolic rewiring happening throughout the estrogen loss-replenish process have not been reported. To investigate the metabolic alteration of sarco-osteoporosis and the possible therapeutical effects of estradiol, 24 mice were randomly divided into sham surgery, ovariectomy (OVX), and estradiol-treated groups. Three-dimensional reconstructions and histopathology examination showed significant bone loss after ovariectomy. Estrogen can well protect against OVX-induced bone loss deterioration. UHPLC-Q-TOF/MS was preformed to profile semi- polar metabolites of skeletal muscle samples from all groups. Metabolomics analysis revealed metabolic rewiring occurred in OVX group, most of which can be reversed by estrogen supplementation. In total, 65 differential metabolites were identified, and pathway analysis revealed that sarco-osteoporosis was related to the alterations in purine metabolism, glycerophospholipid metabolism, arginine biosynthesis, tryptophan metabolism, histidine metabolism, oxidative phosphorylation, and thermogenesis, which provided possible explanations for the metabolic mechanism of sarco-osteoporosis. This study indicates that an UHPLC-Q-TOF/MS-based metabolomics approach can elucidate the metabolic reprogramming mechanisms of sarco-osteoporosis and provide biological evidence of the therapeutical effects of estrogen on sarco-osteoporosis.

## 1. Introduction

Bone and muscle loss alone with aging is considered as a huge threat to loss of independence in later life [1]. Postmenopausal osteoporosis (PMOP) and sarcopenia are common diseases tending to occur simultaneously in elderly people, especially in postmenopausal women [2,3]. They are both associated with significant morbidity and lead to considerable health and social costs [4,5].

PMOP, one of the most concerned health public issues, is characterized by bone loss and microarchitectural deterioration, leaving patients with much higher risk of fractures [6]. In China, 42% women in their 60s have PMOP, as estimated in 2017, and the rate keeps growing with time [7]. Approximately half of postmenopausal women have experienced an osteoporotic fracture during their remaining lifetimes. [8,9]. The medical cost of osteoporotic fracture in China is expected to increase to 264.7 billion US dollars in 2050 [10]. Besides, as a progressive and generalized skeletal muscle disorder, sarcopenia involves the accelerated loss of muscle mass and function that is associated with adverse health consequences including falls, functional decline, frailty, impaired quality of life, and increased health-care costs [4,10,11,12,13,14]. Furthermore, in common with hip and vertebral fractures, a decline in muscle health has also been shown to predict future mortality from middle-age into later life [15].

In addition to the similar population in which they occur, there is also growing evidence of tight links between PMOP and sarcopenia. Postmenopausal osteoporosis is induced by bone loss where bone resorption exceeds bone formation, generally thought to be related to estrogen deficiency. Besides bone, estrogen receptors (ERs) are also found in skeletal muscles [16], and the decline in estrogen level in menopausal women is assumed to be associated with sarcopenia and frailty [16,17], indicating that there might be inherent connections between skeletal muscle cell metabolism and bone anabolism induced by estrogen replacement therapy (ERT) [16].

During the last decade, bone and muscle tissues have increasingly drawn attention worldwide. Growing evidence has shown that skeletal muscles significantly effect bone phenotypes [18,19,20,21] and the ‘bone–muscle’ unit is the site of privileged exchanges, where the two tissues communicate via paracrine and endocrine signals to coordinate their development and adapt their response to loading and injury from embryologic stages to involution [22,23]. As a kind of endocrine tissue attached directly to the bone, skeletal muscle applied load to the bone results in a both mechanical and biological couple [24]. On one hand, physical activity [25] and muscle loss [18], as well as experimental therapy on skeletal muscles [16], stimulate bone anabolism and bone catabolism in numerous ways. On the other hand, physical action interferes with the metabolism of skeletal muscle cells. Thus, skeletal muscle cells influence bone homeostasis in an endocrine way [26]. Taking these factors into consideration, sarcopenia has some potential metabolic connections with osteoporosis, especially in the absence of estrogen.

**Figure 1:**
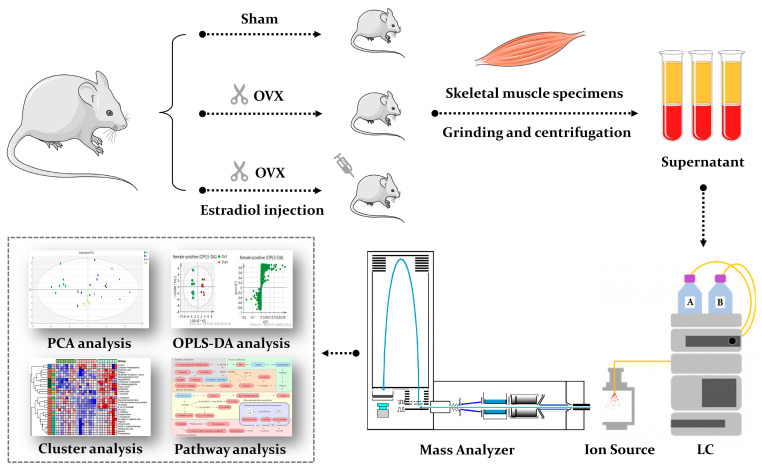
A schematic diagram illustrating the experimental design. We performed an untargeted metabolomics profiling and pathway analysis on skeletal muscle samples of the three groups (sham surgery, ovariectomy, and estradiol-treated group).

Metabolomics refers to a systematic and comprehensive profiling of low molecular weight metabolites from cells, biological fluids and tissues [27], which is a helpful tool that can reveal dynamic changes closest to the phenotype [28]. Monitoring dynamic metabolic changes enables the screening of potential biomarkers or therapeutic targets related to diseases [29]. In recent years, metabolomics has gained growing popularity in the orthopedic field, including osteonecrosis, osteoarthritis, bone tumors, and osteoporosis [30,31]. However, the application of metabolomics in sarco-osteoporosis is scarce. Most of the previous studies on PMOP are mainly limited to plasma samples [32,33]. Until now, metabolomics studies concerning metabolic rewiring occurring throughout the estrogen loss-replenish process in skeletal muscle have not been reported, although a few reports have been published in terms of the specific metabolic effects of sarcopenia on postmenopausal osteoporosis [34,35]. To fill in the gap, we performed an untargeted metabolomics profiling and pathway analysis on skeletal muscle samples of the three groups (sham surgery, ovariectomy, and estradiol-treated group), aiming to reveal the metabolic alterations in sarco-osteoporosis, and to investigate the potential therapeutical effects of E2. Our results demonstrate the differential metabolites and provide a preliminary molecular explanation based on pathway analysis (Figure 1). Thus, our study not only enhances our understanding of the molecular mechanisms of sarco-osteoporosis, but provides a biological basis for estrogen therapy of musculoskeletal degenerative disease.

## 2. Materials and Methods

### 2.1. Chemicals and Reagents

For LC-MS, methanol (HPLC grade), acetonitrile (HPLC grade), formic acid (HPLC grade), and 2-chloro-L-phenylalanine (as an internal standard) were purchased from Sigma. (Saint Louis, MO, USA). Ultrapure water was produced using the Milli-Q system (Millipore, Bedford, MA, USA).

Animal treatment took corn oil (GLBIO, Montclair, CA, USA) as well as estradiol (GLBIO, Montclair, CA, USA) into application. The low limb samples used for micro computed tomography (Micro-CT) and histopathology examinations were preserved applying 80% paraformaldehyde stationary liquid (Labgic Technology, Beijing, China).

In the bone tissue decalcification process, EDTA-2Na and sodium hydroxide were purchased from Sinopharm Chemical Reagent Co., Ltd., Shanghai, China. For hematoxylin-eosin (HE) staining, tartrate-resistant acid phosphatase (TRAP) staining and alkaline phosphatase (ALP) staining, anhydrous ethanol was purchased from Sinopharm Chemical Reagent Co., Ltd. (Shanghai, China). The environmentally friendly dewaxing agent, transparent agent and sealed tablet were purchased from Tonsen tech, Wuhan, China. The histopathological staining kits were purchased from Biossci (Beijing, China) for HE staining (model BP092), TRAP staining (model BP088), and ALP (Model BP090).

### 2.2. Animals and Treatment

Purchased from Shanghai SLAC laboratory Animal Co. Ltd., 24 nine-week-old wild-type female C57/LB6 mice were placed in an adaptive environment after one week of housing under controlled temperature (22–24 °C) and humidity (50–60%) and a 12 h light/dark cycle, with free access to water and food. They were then randomized into three groups: sham (Sham) group, ovariectomy (OVX) group, and estradiol-treated (E2) group. Each group contained eight mice.

After adaptive feeding, mice in the OVX and E2 groups were put under anesthesia with chloral hydrate and ovariectomized in a specific pathogen-free animal laboratory. In the meantime, the Sham group received the sham procedure involving anaesthesia, skin incision and suturing. After one week of recovery, the E2 group received estradiol treatment at a dose of 0.1 mg/kg every two days. After six weeks’ experiment, all mice were sacrificed. Both sides of the lower limbs were harvested within 10 min. Skeletal muscles were obtained from one side of the lower limbs with scissors and then immediately stored in the refrigerator at −80 °C before the samples were pre-treated. The rest of the lower limbs (one side with and another without skeletal muscle) were transferred into 75% ethanol after being immersed in 80% paraformaldehyde stationary liquid for 24 h. All animal experiments and models were approved and performed in accordance with the guidelines of the Animal Care Committee of Shanghai University and the Guide for the Care (Approval Code: ECSHU 2021-196) and Use of Laboratory Animals of the National Institute Health (USA). All animals were housed in a temperature-controlled environment of 22–25 °C with a 12 h light/dark cycle and fed standard rodent chow and water ad libitum.

### 2.3. Sample Preparation

Each weighted sample of skeletal muscle (about 20 ± 1 mg, accurately) was mixed with 500 μL methanol containing 5 μg/mL 2-chloro-L-phenylalanine as internal standard. To carry out homogenate and protein precipitation, mixtures were put into a high-throughput tissue grinder (Tissuelyser-24, Jingxin, Shanghai, China) at 60 Hz for 90 s. Subsequently, centrifugation was applied to samples in 12,000 rpm, 4 °C for 15 min. A total of 100 μL of supernatant of each sample was obtained for further analysis. Pooled quality control (QC) was generated by mixing aliquots (10 μL) of each supernatant. All operations were carried out at −4 °C on ice [33].

### 2.4. Micro-Computed Tomography and Histological Assessment

For Micro-Computed Tomography (micro-CT) assessment, Skyscan 1275 high-resolution micro-CT scanner (Bruker, Billerica, MA, USA) was adapted for three-dimensional reconstructions of femur. Image acquisition was carried out at a voltage of 50 kV, a current of 60 μA, and isotropic resolution of 18 mm. A 0.5–0.75 mm thick aluminum filter was used for beam-hardening reduction. Quantitative morphometric analyses were performed and bone parameters were analyzed for femur samples, including bone mineral density (BMD, g/cm^2^), bone volume/total volume (BV/TV), bone surface to total volume ratio (BS/TV, mm^−1^), and trabecular number (Tb.N, mm^−1^).

After micro-CT scan, the fixed bone samples were decalcified in 10% EDTA for 2 weeks, and then embedded in paraffin blocks for sectioning into 5 mm thick sections. Sections went through HE, TRAP staining for pathology analysis and imaged at 5X and 20X magnification under a light microscope (Nikon, Tokyo, Japan).

### 2.5. UHPLC-Q-TOF/MS Metabolomics Analysis

For UHPLC-Q-TOF/MS analysis, we used Agilent 1290 Infinity LC system coupled with Agilent 6545 accurate mass quadrupole time-of-flight (Q-TOF) mass spectrometer (Agilent, Lexington, MA, USA). Chromatographic column was Waters ACQUITY UPLC HSS T3 analytical column (2.1 mm × 100 mm, 1.8 μm, Waters, Milford, Massachusetts) at 40 °C. The sample chamber temperature was 4 °C. The mobile phase in this experiment was acetonitrile (0.1% formic acid) (eluent A) and water (eluent B) (VH2O: VACN = 95:5, 0.1% formic acid). The flow rate was 0.4 L/min, the injection volume was 5 μL. The real samples were randomized before measurement. QC samples were injected between the real samples after every eight runs. Elution gradient was set in 0–2 min: 0–0% (A); 2–5 min: 0–50% (A); 5–13 min: 50–85% (A); 13–14 min: 85–95% (A); 14–15 min: 95% (A). Both positive and negative ion modes were selected for mass spectrometric conditions. The key parameters were set as follows: mass range was set between 100 and 1100 m/z; fragmentation voltage was set as 120 V; gas temperature was set at 350 °C; the flow rate of drying gas was 11 L/min; nebulizer was set as 45 psig, The capillary voltage was 3.5 kV in positive ion mode and 3.2 kV in negative ion mode.

### 2.6. Data Processing &

After UPLC-Q-TOF/MS data acquisition, the raw data (.d) was transferred into a general data format (mz.data) by applying Agilent MassHunter workstation software version B.01.04 (Agilent, Lexington, MA, USA). Isotope interference was eliminated, and the intensity threshold was set to 300 to eliminate noise. Next, the open-free XCMS (http://metlin.scrippts.edu/, access date: 2 November 2021) was adapted to process the data from each polarity for peak extraction, comparison, and integration to generate a visual data matrix. There are 5170 features in positive mode and 1417 features in negative mode. Then, only the molecular entities detected in at least 80% of the studied samples were considered for further analyses [36]. After filtering, the features in positive and negative mode were 2805 and 683, respectively.

### 2.7. Statistical Analysis

To normalize all the detected ions, the peak areas of the internal standards in each sample were employed to obtain features’ relative intensity, followed by further normalization using the accurate weight of each skeletal muscle sample. In the next step, three-dimensional data matrices including sample name, retention time, m/z pair and normalized ionic intensities were imported into the SIMCA-P program (version 14.1, Umetrics, Umea, Sweden) for multivariate statistical analysis, including principal component analysis (PCA) and orthogonal projections to latent structures discriminant analysis (OPLS-DA). The statistical significance of mean values were tested using students T-test through SPSS Statistics 26 (IBM, New York, NY, USA). Differences were considered to be significant when P value was less than 0.05.

### 2.8. Pathway Analysis

Metaboanalyst website (http://www.metaboanalyst.ca/, access date: 7 November 2021) was used to conduct pathway analysis. At first, the differential metabolites were imported into this website and matched with HMDB, PubChem and KEGG databases (the quality error tolerance is less than 15ppm), then classified according to KEGG links. Based on the obtained enriched KEGG pathways, the pathway network was then constructed manually, and some important pathways were highlighted.

## 3. Results

### 3.1. Body, Tissue Weight and Clinical Observation

During the experimental period, animals were weighted once a week (Figure S1). The thigh diameter and uterus weight of each animal were measured on the day of the dissection. No significant differences were observed in the body weight or thigh diameter of the animals (Figure S2). The uterus weight of mice in the OVX group was significantly lower than that of the Sham and E2 group, and the mice in the E2 group had the highest average uterus weight, even higher than those in the Sham group (*p* < 0.05, Figure S3).

### 3.2. Micro-Computed Tomography Assessment

**Figure 2:**
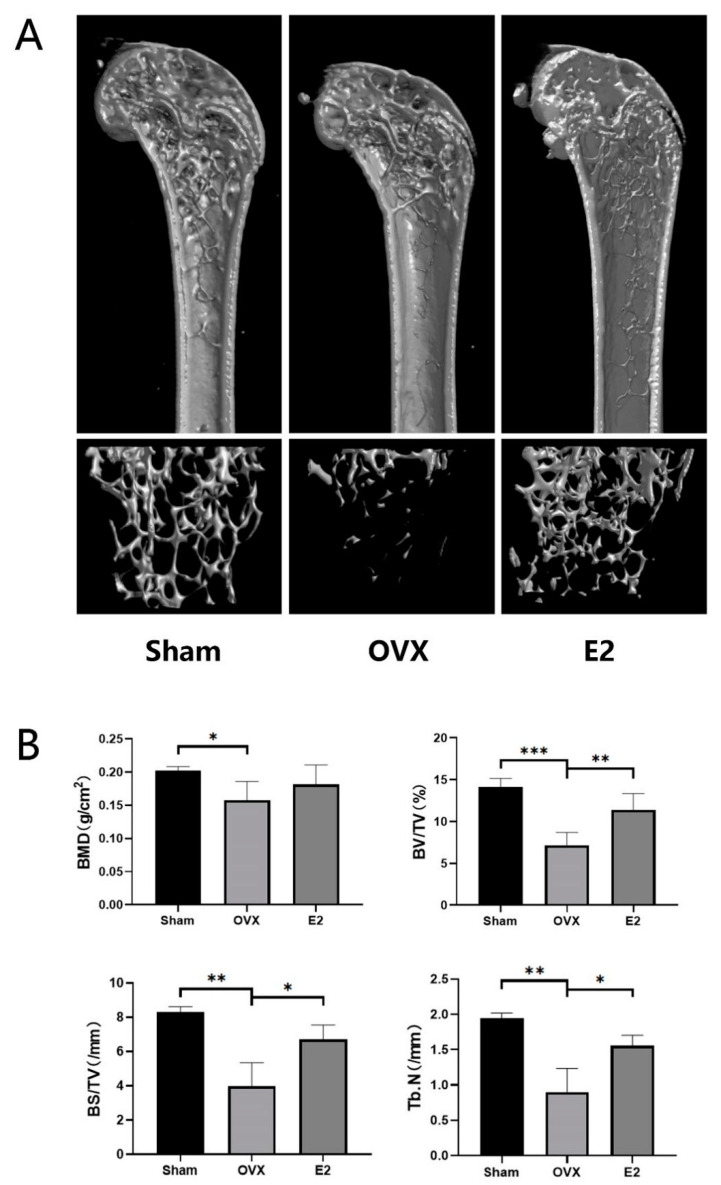
Estradiol (E2) prevents bone loss in ovariectomized (OVX) mice in vivo. (**A**) Representative 3D Micro-CT reconstructions of mouse femur from sham (PBS injection), OVX (PBS injection), and E2 (OVX with 0.1 mg/kg estradiol). (**B**) Quantitative bone morphometric parameters of bone mineral density (BMD, g/cm^2^), bone volume to total tissue volume (BV/TV), bone surface to tissue volume (BS/TV, mm−1), and trabecular number (Tb.N., mm−1) were measured. Values presented as the mean ± standard deviation (n = 3); * *p* < 0.05, ** *p* < 0.01, *** *p* < 0.001.

Quantitative bone morphometric parameters of bone mineral density (BMD, g/cm^2^), bone volume to total tissue volume (BV/TV), bone surface to tissue volume (BS/TV, mm^−1^), and trabecular number (Tb.N., mm^−1^) were measured. Three-dimensional reconstructions of the femoral bone tissue showed a significant reduction in bone volume, trabecular bone loss and all the measured quantitative bone morphometric parameters after bilateral ovariectomy in the OVX group (Figure 2A,B). On the contrary, treatment with estrogen can restore OVX-induced bone loss and trabecular bone deterioration and showed an increasing trend of all the measurements (Figure 2A,B).

### 3.3. Skeletal Muscle Histopathology

**Figure 3:**
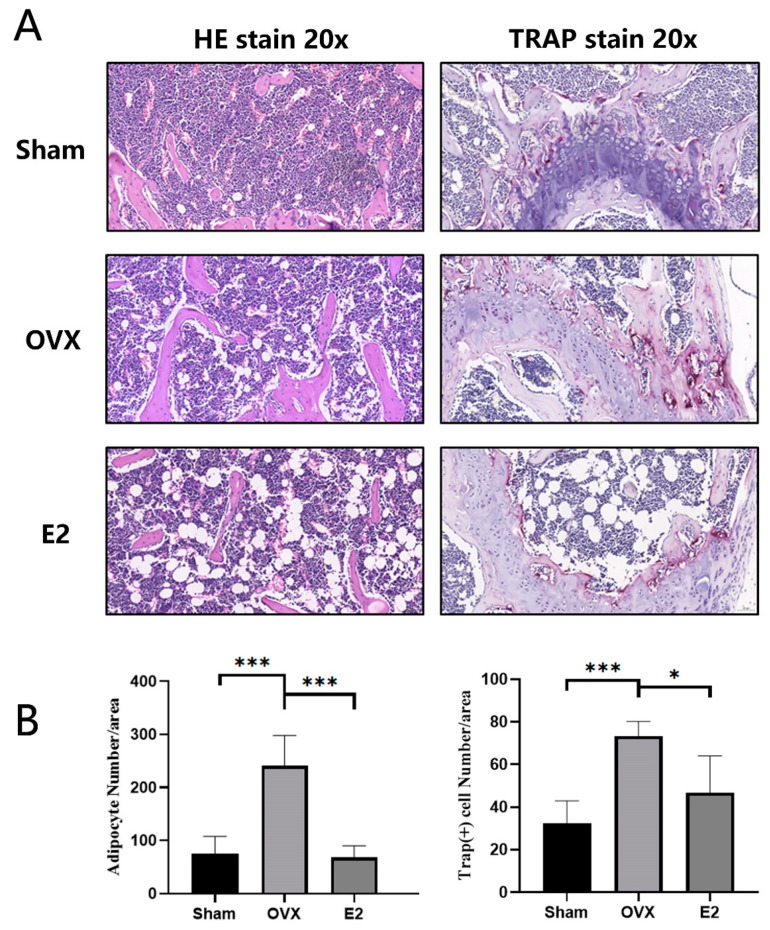
Photomicrographs of femurs. (**A**) Representative histological assessment of mouse femur sections stained for H&E and TRAP activity (20X magnification). (**B**) Quantitative assessment of the total number of adipocytes and TRAP+ cells per bone surface were conducted. Values presented as the mean ± standard deviation (n = 3); * *p* < 0.05, *** *p* < 0.001.

HE staining of femur demonstrated that, compared with the Sham group, the OVX group showed a significant increase in adipocytes and severe myodemia. In addition, we observed significant protective effect in the E2 group with fewer adipocytes and alleviated myodemia (Figure 3A,B). The same trend was seen in the TRAP staining of the femur sections, demonstrating significant enhancement in the total number of TRAP+ osteoclasts and the number of osteoclasts lining the trabecular bone surface in OVX group and the total number of TRAP+ osteoclasts decreased in E2 group at the same time (Figure 3A,B).

### 3.4. Metabolomics Profiling Analysis

Applying the optimized LC-MS method mentioned above, the raw metabolomic data of the mouse skeletal muscle was obtained. To demonstrate the total ions detected in the measurement, the representative total ion chromatograms (TIC) in both positive and negative ion modes are shown in Figure S4.

The normalized data were introduced to SIMCA-P 14.1 (Umetrics, Malmo, Sweden). The unsupervised PCA-X analysis was adapted to assess the stability of data obtained from the sample between QC samples and the real samples. As shown in Figure S5, all the QC samples are clustered together in both positive and negative ion modes, indicating good system stability.

**Figure 4:**
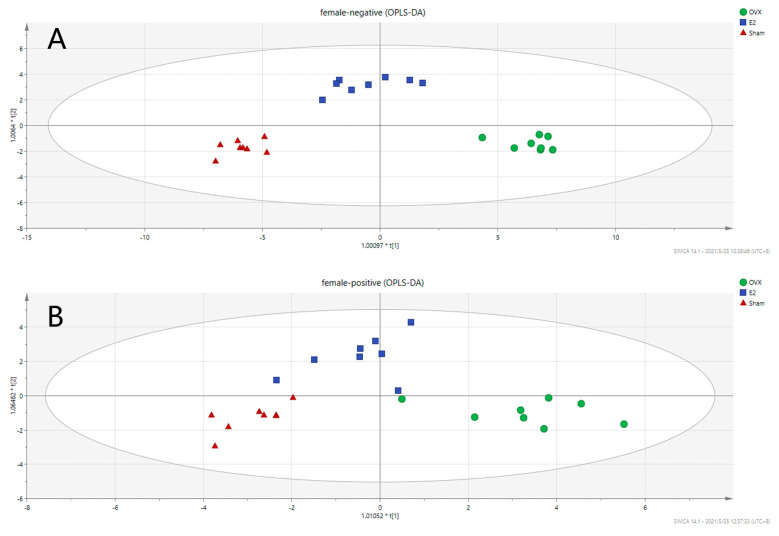
Score Scatter Plots for OPLS-DA analysis in (**A**) negative mode and (**B**) positive mode.

To investigate the metabolic differences among the Sham, OVX, and E2 groups, OPLS-DA analysis was conducted, and the results are illustrated in Figure 4 (Positive mode: R2X = 0.667, R2Y = 0.868, Q2 = 0.727; Negative mode: R2X = 0.781, R2Y = 0.94, Q2 = 0.76). OPLS is a regression modeling method of multiple dependent variables to multiple independent variables. It can recognize the most constructive ions to the clustering of samples as well as remove the irrelevant data variation from the data set [37]. Score plots from OPLS-DA analysis indicate that there exists a separation among the Sham, OVX and E2 groups. The samples in the same group showed a good clustering trend. Moreover, there was a tendency of E2 group approaching to the Sham group comparing to OVX group, indicating that E2 treatment partially restored the metabolic changes induced by OVX surgery.

**Figure 5:**
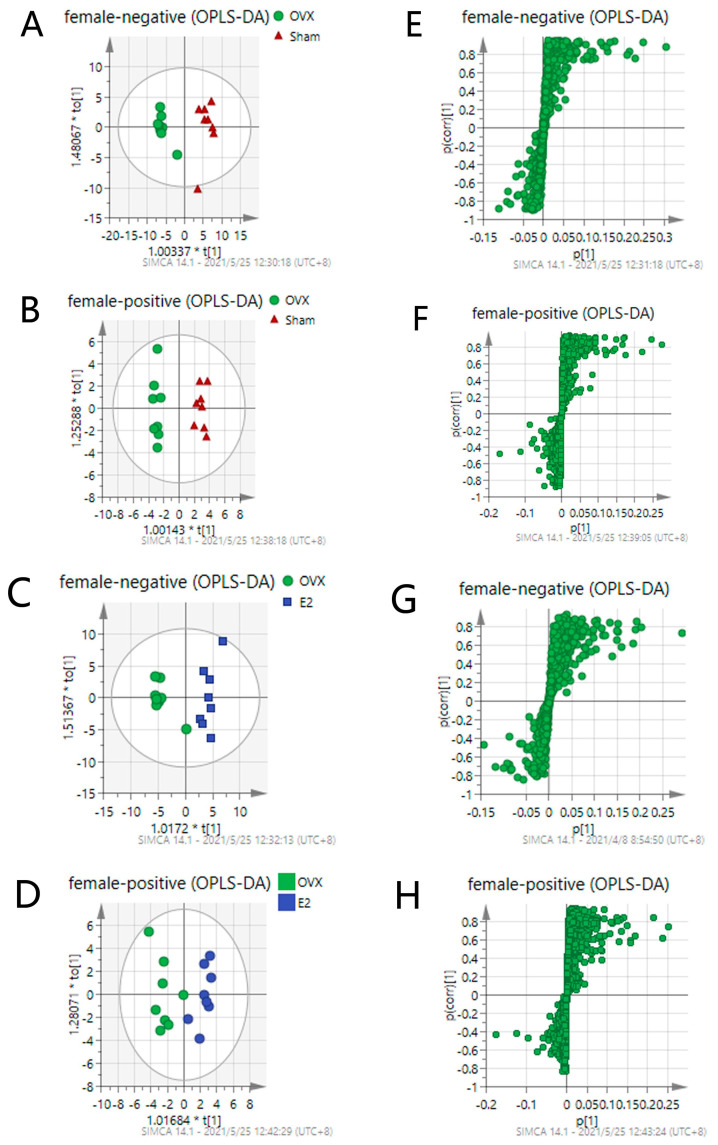
OPLS-DA score plots of Sham, OVX and E2 group (**A**–**D**) and their corresponding S-plot (**E**–**H**). Promising degree of fitting and predictive ability make it reliable to screen the differential variables between groups: (**A**,**E**) OVX vs. Sham in negative mode; (**B**,**F**) OVX vs. Sham in positive mode; (**C**,**G**) OVX vs. E2 in negative mode; (**D**,**H**) OVX vs. E2 in positive mode. S-plot shown in (**E**–**H**) is composed of ions separating according to the contribution of the ion on the difference between two groups. Each ion is presented as one point in the s-plot. The further the point is to the original point, the more impact the ions have on the differentiation of two groups and thus has the bigger VIP value.

Pairwise OPLS-DA analyses between the Sham, OVX and E2 groups characterize the differences between each pair of the groups. Figure 5 shows that there is an obvious clustering relationship among these three groups. Promising degrees of fitting and predictive ability make it reliable to screen the differential variables between groups.

S-plot shown in Figure 5E–H is composed of ions separating according to the contribution of the ion on the differences between each pair of the groups. Each ion is presented as one point in the S-plot. The further the point is located to the original point, the more impact the ion has on the differentiation of two groups and, thus, has a bigger VIP value. Applying the independent sample t-test as an assessment of the statistical significance, the *p* values can also be attained. To select the important ions differentiating the Sham and the OVX group, and the OVX and the E2 group, only the ions with *p* < 0.05 and VIP > 1 were selected. There were 33 and 35 differential metabolites identified in positive and negative mode, respectively. Differential metabolites between the Sham and the OVX group reflect the metabolic changes that occurred after the ovariectomy procedure was performed on the mice. Table 1 shows the identified 65 differential metabolites in comparison between the Sham and OVX groups. Meanwhile, it was also meaningful to observe whether these metabolites can be reversed by E2 treatment when compared to the OVX and E2 groups, providing a comprehensive understanding of the metabolic changes occurring in the estrogen-loss and -regain process.

**Figure 6:**
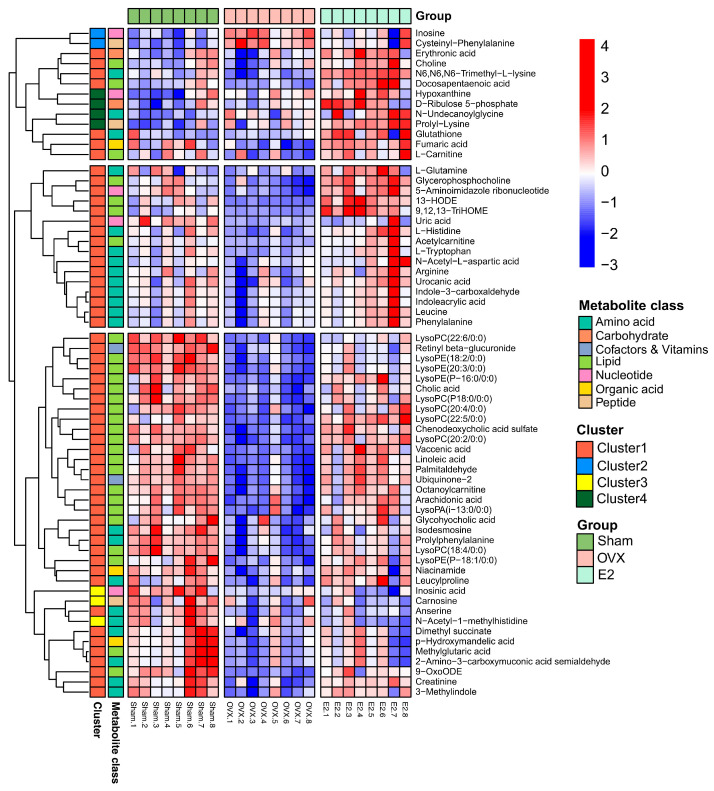
The clustering heatmap of Sham, OVX and E2 group based on the 65 differential metabolites in skeletal muscle.

**Figure 7:**
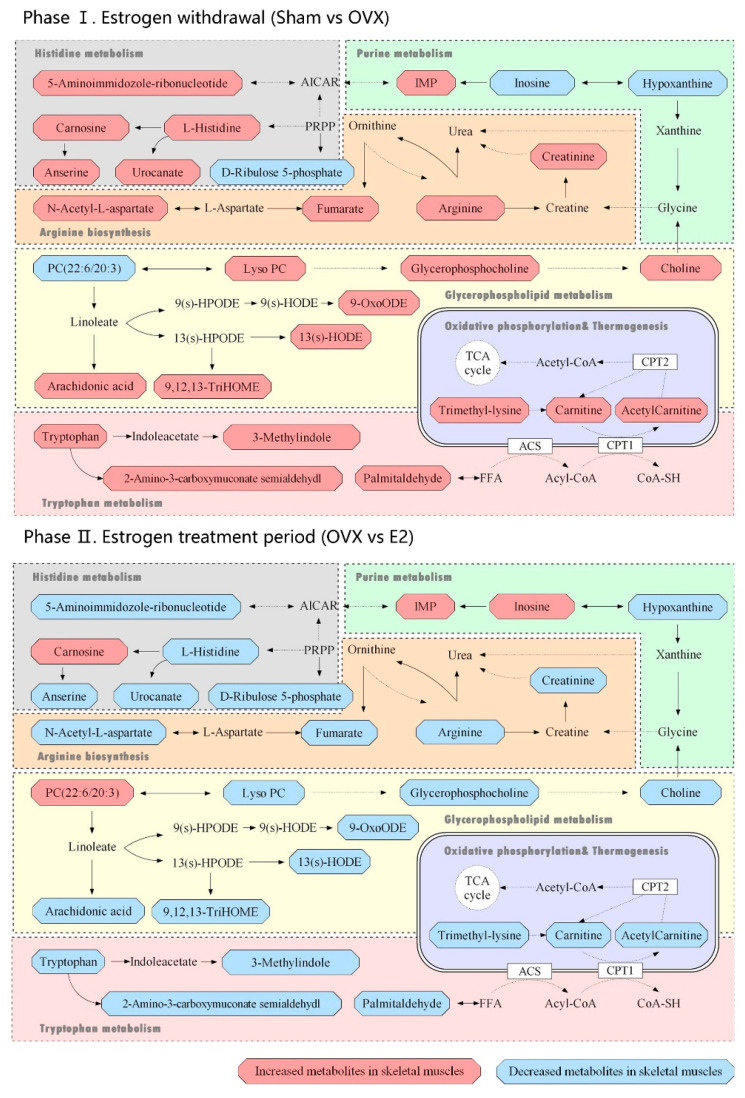
Metabolic pathways related to the differential metabolites identified in the Sham, OVX and E2 group. Phase Ⅰ: Estrogen withdrawal (Sham vs. OVX); Phase Ⅱ. Estrogen treatment period (OVX vs. E2).

The heatmap (Figure 6) displays the dramatic changes of the 65 differential metabolites among the Sham, OVX and E2 groups. According to our results, we divided the treatment of sarcopenia and osteoporosis with estrogen into two phases: estrogen withdrawal (Sham vs. OVX) and treatment period (OVX vs. E2). A total of 58 metabolites demonstrated a reversal of trend during the treatment period and seven metabolites showed a consistent trend in both periods (N-acetyl-1-methylhistidine, carnosine, inosinic acid, hypoxanthine, N-undecanoylglycine, prolyl-lysine, D-ribulose 5-phosphate). The metabolites can automatically cluster into four groups, which were described as Cluster 1-4 in the heatmap. Cluster 1 included the down-and-up regulated components, which meant that compared with Sham group, the level of these metabolites decreased in the OVX group and were up-regulated in the E2 group. Similarly, Cluster 2 included up-and-down regulated components, Cluster 3 involved down-and-down regulated components and Cluster 4 involved up-and-up regulated components. The pathway information on these 65 differential metabolites based on the KEGG pathway database (http://www.genome.jp/kegg/, access date: 7 November 2021) is summarized in Table 1. By pathway enrichment analysis, we determined that several metabolic pathways are significantly enriched (Figure 7), including purine metabolism, glycerophospholipid metabolism, arginine biosynthesis, tryptophan metabolism, histidine metabolism, arachidonic acid metabolism, oxidative phosphorylation, and thermogenesis, indicating these pathways may be involved in the molecular mechanisms of PMOP. Based on the metabolite chemical reactions in KEGG, we constructed a metabolic network for the 65 differential metabolites (Figure 7), suggesting an intricate regulation system underlying the complex pathology of PMOP and sarcopenia. Strikingly, we found that among these 65 significantly differential metabolites, 58 of them can be restored by E2 treatment, indicating promising clinical application of E2 in the musculoskeletal degeneration field.

## 4. Discussion

Approximately half of postmenopausal women have an osteoporosis-related fracture during their remaining lifetime, which has become a serious global health problem [9]. Postmenopausal osteoporosis is generally thought to be caused by a deficiency of estrogen. As a steroid hormone, estrogen is secreted primarily from the ovaries. It has been proved that estrogen plays various important roles in non-reproductive organs or tissues, including skeletal, immune, cardiovascular, and central nervous systems as well as in their metabolism [38,39]. The low estrogen state experienced by women following the menopause or bilateral oophorectomy affects the physiological functions of these non-reproductive systems [40,41]. On the one hand, the decrease in estrogen level associated with athletic amenorrhea can lead to bone weakness and fatigue fracture [42,43]. On the other hand, a decline in estrogen level in menopausal women is assumed to be associated with sarcopenia and frailty, therefore it has been suggested that estrogen also regulates muscle mass and function [16,17]. More importantly, it also appears that local factors and metabolites related to altered muscle-bone crosstalk underly the coordinated loss of muscle and bone in these settings. For example, muscle is a source of myokines that can stimulate bone formation and also contribute to bone loss [44,45], whereas bone secretion factors such as osteocalcin and connexin 43 have direct effects on muscle [46,47], meaning that postmenopausal sarcopenia has some potential metabolic connections with osteoporosis. However, very few studies have reported in terms of the specific effects of estrogen deficiency-derived sarcopenia on postmenopausal osteoporosis [34,35]. Therefore, it is of great significance and interest to study this problem and reveal the potential metabolic mechanism.

In this study, an ovariectomized model was successfully built in mice and we applied the UPLC-Q-TOF/ MS-based metabolomics profiling to investigate the metabolic alterations of skeletal muscle tissue in the Sham, OVX and E2 mice. In total, 65 differential metabolites from skeletal muscle tissues were identified by combining the OPLS-DA and t-test. Then, we constructed a metabolic pathway network that clearly showed PMOP, and sarcopenia were related to the alterations involved in the purine metabolism, glycerophospholipid metabolism, arginine biosynthesis, tryptophan metabolism, histidine metabolism, arachidonic acid metabolism, oxidative phosphorylation, and thermogenesis.

Severe lipid pathways were significantly different in the skeletal muscle tissues of the OVX group. Phosphatidylcholine was significantly upregulated in phase Ⅰ and downregulated in phase Ⅱ (Figure 5). Besides, lysophospholipids (LysoPC/LysoPE/LysoPS) showed opposite trends in both phases, which indicates that the deficiency of estrogen resulted in the reduction in the lipid metabolism, including arachidonic acid, choline and lysophospholipids at the point of phosphatidylcholine. This alteration was corrected by estrogen supplementation, which was consistent with the results of other studies [48,49]. It has been reported that in the regeneration defect model, ferroptosis is activated during muscle regeneration with Tfr1 deletion in satellite cells [50]. This event is coupled with labile iron accumulation, unsaturated fatty acid biosynthesis, and decreased expression of anti-ferroptosis biomarkers, which has been recapitulated in rodents’ aged skeletal muscle and human sarcopenia [51,52]. Similarly, we observed fat accumulation, fatty acids transport alterations, such as carnitine down-regulation and lipid metabolism changes. The possible mechanism was in the absence of macrophage Fpn, iron sequestered inside the macrophages can prevent muscle regeneration and activates adipogenesis, leading to fat accumulation [53]. On the other hand, the labile iron accumulation is derived from myoglobin degradation by Hmox1, and additional non-transferrin-bound iron absorption is facilitated by Slc39a14, which exacerbates iron accumulation and oxidative damage [53]. We also detected that glutathione was significantly downregulated, providing the evidence of oxidative damage involved in oxidative phosphorylation and thermogenesis.

Furthermore, carnosine, anserine and L-histidine were detected to decrease in the OVX group and were reversed in the E2 group. In the bone formation process, histidine regulated the plate-shaped hydroxyapatite, which is the main inorganic compound of bone. Chauhan [54] et al. reported that L-histidine can replace large non-collagenous proteins as a modifier of hydroxyapatite. Moreover, a plate-shaped hydroxyapatite-consisted environment provides a better propagate environment for mice osteoblast precursor cells. Therefore, a decline in L-histidine might indicate a signal for a less osteoblast cell number and the regain of L-histidine by E2 treatment reverses the trend. In vitro experience [55], the cytotoxic effect of dipeptides is exerted by inhibiting the DNA synthesis process, which mainly affects the ab initio synthesis of purines and the inhibition of the activities of 5-phosphoribosyl 1-pyrophosphate (PRPP) aminotransferase. For mice accepting the OVX procedure, the reduction in histidine metabolism indicates less activity of PRPP. Since impairment of PRPP level might be the reason for energy metabolism disorder [56], the interrupted energy metabolism in bones is revealed.

Purine metabolism participates in numerous physiological and pathological processes in mammals such as inflammatory response, oxidative stress reaction and cancer [57,58]. As the final product of purine metabolism, uric acid has been proved to be a major factor interrupting the synthesis of dihydroxy vitamin D [59] in the kidneys, and thus interrupts the absorption of intestinal calcium and reduces blood calcium. The significant changes of inosine may reflect the changes in BMD, since it is an accepted biomarker for osteoporosis [60]. As shown in Figure 6, there is a call-back trend of inosine concentration in the E2 group, consistent with the change in BMD level. However, it is of note that the hypoxanthine level remains increasing despite the intervention of ERT. Considering that hypoxanthine is a source of oxidative stress in the vascular system [61], this phenomenon might be an alert for researchers that the oxidation stress from the PMOP process can be permanent if patients take no more treatment other than ERT.

Creatinine is the breakdown product of creatine in muscles. A decrease in creatinine is a sign of disordered energy and amino acid metabolism phenomena [62]. By the metabolomic approach, we observed that the OVX group showed a reduction in creatinine, arginine and other metabolites related to arginine biosynthesis. With the supplement of estrogen, a clear call-back of arginine biosynthesis can be revealed. The decline in arginine biosynthesis level indicates a disability for iron process [63]. At the molecular level, an overdose of iron is generally considered to be toxic for the bone mineralization process, exerting the apoptosis of osteoblasts and hereby reducing mineralization capacity [64,65]. The recovery in both arginine biosynthesis and BMD in the E2 group showed a sign of iron process revival, where a potential chance arose for the reconstruction of bone homeostasis. Under iron balanced circumstances, osteoblasts can be kept away from threats [66] and the osteoclasts number should decrease compared to the iron resorption unbalanced group [67].

As one of the eight essential amino acids in human nutrition, tryptophan plays important roles in several biological processes such as synthesis of 5-hydroxytryptamine and renewal of plasma proteins in animals. In bone homeostasis studies, a decrease in tryptophan is considered a typical biomarker for osteoporosis [68]. Tryptophan metabolism can be divided into two parts: the synthesis of protein and decomposition of enzymes. The hydroxylated product of tryptophan is serotonin (5-HT) [69]. Therefore, a decrease in tryptophan results in a lower level of 5-HT, where the regulatory hormone level is not enough [70] for both skeletal muscle growth and secretion of insulin-like growth factors (IGFs) by the liver [71]. IGFs are a group of peptide hormones with anabolic functions that promote the differentiation of myoblasts and osteogenic tissues [72]. The supplement of estrogen reversed this phenomenon into a relatively normal level.

During the past few years, metabolomics has become an exciting and evolving research area, helping us to overcome challenges that initially confounded analysis. However, there still exists major challenges in identifying metabolites and validating metabolites in human populations. In addition, the most important challenge is to develop and promote workflows for verifying biological meaning in metabolites and to move towards determining the mechanisms of the disease [73,74].

## 5. Conclusions

In this study, we successfully constructed an ovariectomized model to simulate PMOP and sarcopenia in mice, verified via clinical observation, micro-computed tomography assessment and skeletal muscle histopathology examination. Untargeted UPLC-Q-TOF/MS-based metabolomics profiling was applied to comprehensively exhibit the metabolic alterations in skeletal muscle in the Sham, OVX, and E2 treated mice. In total, 65 differential metabolites in skeletal muscle tissue were identified. Pathway analysis revealed that several pathways were potentially related to estrogen deficiency-induced bone and muscle loss, including purine metabolism, glycerophospholipid metabolism, arginine biosynthesis, tryptophan metabolism, histidine metabolism, arachidonic acid metabolism, oxidative phosphorylation, and thermogenesis. These findings demonstrated the metabolic changes of estrogen deficiency-induced bone and muscle loss in mice. Our study will hopefully encourage awareness of the connections between PMOP and sarcopenia by providing both a possible molecular explanation for metabolic rewiring and a molecular basis for estrogen therapy of musculoskeletal degenerative disease. It also demonstrates that an UHPLC-Q-TOFMS-based metabolomics approach is a valuable tool to advance our understanding of PMOP and sarcopenia.

## Figures and Tables

**Table 1:** Differential metabolites related to sarco-osteoporosis and therapeutical effects of estradiol throughout the estrogen loss-replenish process.

No.	m/z	RT (min)	Name	Formula	Adduct	VIP		FC		Pathway	Phase ^#^
						[Sham-OVX]	[OVX—E2]	[Sham/OVX]	[OVX/E2]		
1	104.1068	0.628326	Choline	C5H14NO	M+H	1.99055	4.15666	—	0.761420332220201 *	Glycerophospholipid metabolism	Ⅰ
2	114.0655	0.670794	Creatinine	C4H7N3O	M+H	1.12556	1.20794	1.16838813632129 ***	0.868640449077801 **	Arginine biosynthesis	Ⅰ
3	115.0044	1.133651	Fumaric acid	C4H4O4	M-H	0.823357	1.3535	1.18475649691836 **	0.782460430064511 ***	—	Ⅰ
4	123.056	0.993859	Niacinamide	C6H6N2O	M+H	4.34495	3.07659	1.3146239858077 **	—	Nicotinate and nicotinamide metabolism	Ⅰ
5	128.9597	0.600534	Dimethyldisulfide	C2H6S2	M+Cl	1.33201	1.0461	1.68360931432131 ***	0.748896335151599 *	—	Ⅰ
6	132.08	0.671339	3-Methylindole	C9H9N	M+H	8.14758	7.73113	1.11695054898848 **	0.915561301482534 *	Tryptophan metabolism	Ⅰ
7	132.1006	1.212927	Leucine	C6H13NO2	M+H	5.14486	11.1368	—	0.656558924361863 *	—	Ⅰ
8	137.0447	1.007996	Erythronic acid	C4H8O5	M+H	2.11715	5.11092	—	0.842216410457762 *	—	Ⅰ
9	145.0649	0.629002	L-Glutamine	C10H10O	M-H	0.324981	1.64018	—	0.807916416097675 *	Arginine biosynthesis	Ⅰ
10	145.051	1.392685	Methylglutaric acid	C6H10O4	M-H	2.44631	1.58801	1.73862365343816 **	—	—	Ⅰ
11	146.0592	3.753873	Indole-3-carboxaldehyde	C9H7NO	M+H	0.716426	1.09405	—	0.642949508463612 *	Purine metabolism	Ⅰ
12	154.0622	0.595069	L-Histidine	C6H9N3O2	M-H	0.746679	1.33778	—	0.687650886091606 **	Histidine metabolism	Ⅰ
13	156.0757	0.580905	Urocanic acid	C6H6N2O2	M+NH4	0.891396	1.57463	—	0.76801224287754 *	Histidine metabolism	Ⅰ
14	162.1134	0.647544	L-Carnitine	C7H15NO3	M+H	1.39599	2.24982	—	0.814676279423792 *	Oxidative phosphorylation & Thermogenesis	Ⅰ
15	166.0851	2.001319	Phenylalanine	C9H8O2	M+NH4	3.40204	7.36224	—	0.66880554095327 *	—	Ⅰ
16	167.0195	0.890411	Uric acid	C5H4N4O3	M-H	1.5676	1.53901	1.86295694809978 *	—	Purine metabolism	Ⅰ
17	175.1182	0.605419	Arginine	C6H14N4O2	M+H	0.950214	1.63368	—	0.697768957488221 *	Arginine biosynthesis	Ⅰ
18	176.0562	1.216735	N-Acetyl-L-aspartic acid	C6H9NO5	M+H	0.457301	1.03731	—	0.591565318586933 *	Arginine biosynthesis	Ⅰ
19	188.0696	3.753191	Indoleacrylic acid	C11H9NO2	M+H	1.96072	3.12121	—	0.633975178007676 *	—	Ⅰ
20	189.1586	0.587062	N6, N6, N6-Trimethyl-L-lysine	C9H20N2O2	M+H	0.581521	1.1139	1.26553165740387 *	0.598635929115935 ***	Oxidative phosphorylation & Thermogenesis	Ⅰ
21	202.1095	4.602232	Acetylcarnitine	C9H17NO4	M-H	0.723706	1.3691	—	0.393454866839646 *	Oxidative phosphorylation & Thermogenesis	Ⅰ
22	203.0837	3.794141	L-Tryptophan	C11H12N2O2	M-H	0.688977	1.22077	—	0.705435116239765 *	Tryptophan metabolism	Ⅰ
23	213.0393	1.391897	p-Hydroxymandelic acid	C8H8O4	M+FA-H	1.01894	0.580048	1.60962362078635 *	—	—	Ⅰ
24	229.1531	1.021603	Leucylproline	C11H20N2O3	M+H	1.42508	1.93097	1.22695989785742 *	0.779746081798664 **	—	Ⅰ
25	230.0297	1.39159	2-Amino-3-carboxymuconic acid semialdehyde	C7H7NO5	M+FA-H	1.40212	0.854046	1.76871679389686 **	—	Tryptophan metabolism	Ⅰ
26	239.116	0.584029	Anserine	C10H16N4O3	M-H	1.45976	0.788336	1.29276359029791 **	—	Histidine metabolism	Ⅰ
27	258.1109	0.638932	Glycerophosphocholine	C8H20NO6P	M+H	1.37042	2.06634	1.83918725529827 **	0.38912024015719 ***	Glycerophospholipid metabolism	Ⅰ
28	263.2338	13.50023	Palmitaldehyde	C16H32O	M+Na	1.0577	1.2071	2.1074416504338 * **	0.500126885803229 ***	Tryptophan metabolism	Ⅰ
29	279.2353	13.49943	Linoleic acid	C18H32O2	M-H	2.18294	2.44137	2.25020157276134***	0.490801930932198 ***	—	Ⅰ
30	280.1653	8.966165	Prolylphenylalanine	C14H18N2O3	M+NH4	1.04127	0.965697	1.42805455591819 ***	0.774828595167275 **	—	Ⅰ
31	281.2507	14.64251	Vaccenic acid	C18H34O2	M-H	0.896885	1.09955	2.1528646984969 ***	0.461301521076128 ***	—	Ⅰ
32	293.2146	9.545482	9-OxoODE	C18H30O3	M-H	1.80771	1.77528	2.28277988282034 ***	0.551308194208161 **	Glycerophospholipid metabolism	Ⅰ
33	295.2301	9.298135	13-HODE	C18H32O3	M-H	0.948831	1.64456	1.93721337344917 **	0.357258751691486 ***	Glycerophospholipid metabolism	Ⅰ
34	296.0646	0.617891	5-Aminoimidazole ribonucleotide	C8H14N3O7P	M+H	0.924232	1.3571	1.88084682781242 **	0.404760064989924 ***	Histidine metabolite	Ⅰ
35	303.2358	13.31774	Arachidonic acid	C20H32O2	M-H	3.12025	3.5239	1.53409019367542 ***	0.706526468814323 **	Glycerophospholipid metabolism	Ⅰ
36	305.2444	13.31633	Octanoylcarnitine	C15H29NO4	M+NH4	2.1425	2.39263	1.74294985577128 ***	0.617880771536116 ***	—	Ⅰ
37	306.0736	0.853002	Glutathione	C10H17N3O6S	M-H	0.271999	1.8032	—	0.613780322915892 *	Cysteine and methionine metabolism	Ⅰ
38	319.191	13.50041	Ubiquinone-2	C19H26O4	M+H	0.874951	1.0324	1.74238563302715 ***	0.582455077036921 ***	—	Ⅰ
39	329.2339	5.829618	9,12,13-TriHOME	C18H34O5	M-H	0.454282	1.1416	—	0.374493955652705 ***	Glycerophospholipid metabolism	Ⅰ
40	329.2514	14.05921	Docosapentaenoic acid	C22H34O2	M-H	0.498006	1.22922	—	0.473442901456016 ***	Biosynthesis of unsaturated fatty acids	Ⅰ
41	403.1622	13.30849	LysoPA(i-13:0/0:0)	C16H33O7P	M+Cl	2.1169	2.45337	1.5786366934321 **	0.669220463950303 **	—	Ⅰ
42	436.2879	9.421978	LysoPE(P-16:0/0:0)	C22H43NO5	M+Cl	1.63689	1.90884	1.83811965532063 ***	0.55218775697055 ***	—	Ⅰ
43	453.2865	8.904551	Cholic acid	C24H40O5	M+FA-H	1.38344	1.32711	1.7272126612545 ***	0.692868741397353 *	Primary bile acid biosynthesis	Ⅰ
44	462.3036	9.894062	LysoPE(P-18:1/0:0)	C23H46NO6P	M-H	1.02737	1.11653	1.57754658465202 **	0.680272204730959 *	—	Ⅰ
45	466.3153	11.28517	Glycohyocholic acid	C26H43NO6	M+H	1.19529	1.21009	1.59921132406263 *	—	—	Ⅰ
46	480.2975	9.266177	Retinyl beta-glucuronide	C26H38O7	M+NH4	2.9821	2.05286	1.76295122148748 ***	0.773558552128259 *	—	Ⅰ
47	481.3185	10.71418	LysoPE(18:0/0:0)	C26H44O5	M+FA-H	2.94576	2.47977	1.92511114432689 ***	—	—	Ⅰ
48	490.2786	7.467329	Chenodeoxycholic acid sulfate	C24H40O7S	M+NH4	0.982727	1.04848	2.17942495354643 ***	0.478755397066275 ***	—	Ⅰ
49	516.3049	8.961922	LysoPC(18:4/0:0)	C26H46NO7P	M-H	1.56594	1.35042	2.10833476650201 ***	0.597857675967578 **	Glycerophospholipid metabolism	Ⅰ
50	520.2712	8.904493	LysoPS(18:2/0:0)	C24H44NO9P	M-H	1.03408	1.04804	1.64204279449899 ***	0.699072746882726 *	—	Ⅰ
51	526.2842	8.213898	Isodesmosine	C24H40N5O8	M+H	5.73742	4.33894	1.41467979845622 ***	—	—	Ⅰ
52	548.3674	9.647121	LysoPC(20:2/0:0)	C28H54NO7P	M+H	1.09737	1.19136	2.49636956071126 ***	0.414483299250478 ***	Glycerophospholipid metabolism	Ⅰ
53	548.3033	10.71418	LysoPE(20:3/0:0)	C25H46NO7P	M+FA-H	1.70686	1.51257	1.62794342299198 ***	—	—	Ⅰ
54	588.3399	8.285756	LysoPC(20:4/0:0)	C32H48O7	M+FA-H	1.07686	1.18063	1.32460166924025 ***	0.788136582237281 **	—	Ⅰ
55	612.3412	8.276272	LysoPC(22:6/0:0)	C37H47NO4	M+FA-H	1.49253	1.17802	1.82482512793769 ***	0.750351737131499 **	—	Ⅰ
56	614.346	9.084809	LysoPC (22:5/0:0)	C30H52NO7P	M+FA-H	0.894325	1.31495	1.43993246105778 ***	0.5970624600792 ***	Glycerophospholipid metabolism	Ⅰ
1	267.0714	0.707363	Inosine	C10H12N4O5	M-H	2.00802	2.33534	0.770100006297074 **	—	Purine metabolism	Ⅱ
2	291.0762	0.72853	Cysteinyl-Phenylalanine	C12H16N2O3S	M+Na	2.21001	1.71329	0.714621327035955 ***	—	—	Ⅱ
1	212.1008	0.573274	N-Acetyl-1-methylhistidine	C9H10N2O3	M+NH4	0.563681	0.399834	—	—	—	Ⅲ
2	225.1001	0.583991	Carnosine	C9H14N4O3	M-H	0.737296	0.212796	—	—	Histidine metabolism	Ⅲ
3	349.0534	1.006389	Inosinic acid	C10H13N4O8P	M+H	2.43973	0.20068	1.624576293826 **	—	Purine metabolism	Ⅲ
1	135.0314	1.011678	Hypoxanthine	C5H4N4O	M-H	0.639457	0.931754	—	—	Purine metabolism	Ⅳ
2	242.1778	5.770676	N-Undecanoylglycine	C13H25NO3	M-H	0.967668	0.53091	—	—	—	Ⅳ
3	266.1456	6.31293	Prolyl-Lysine	C11H21N3O3	M+Na	0.714918	0.989698	—	0.807036290414128 *		Ⅳ
4	275.0193	0.695852	D-Ribulose 5-phosphate	C5H11O8P	M+FA-H	0.304399	0.701141	—	—	—	Ⅳ

*: 0.05 > *p* > 0.001, **: 0.001 > *p* > 0.0001, ***: 0.0001 > *p*. “—”: The difference was not statistically significant or no meaningful pathway was determined. ^#^: Phase: Ⅰ: down-and-up regulated components, Ⅱ: up-and-down regulated components, Ⅲ: down-and-down regulated components, Ⅳ: up-and-up regulated components.

## Supplementary Materials

The following supporting information can be downloaded at: https://www.mdpi.com/article/10.3390/biom12010041/s1. Figure S1. Body weight of mice in Sham, OVX and E2 group was recorded (n = 6). No significant differences were observed (*p* > 0.1). Figure S2. Thigh diameter of mice in Sham, OVX and E2 group was measured (n = 6). No significant differences were observed (*p* > 0.05). Figure S3. The average uterus weight of mice in Sham, OVX and E2 group was measured (n = 6, *p* < 0.05). Figure S4. Representative total ion chromatograms of UPLC-Q-TOF/MS (A) TIC of Sham group in positive mode (B) TIC of Sham group in negative mode (C) TIC of OVX group in positive mode (D) TIC of OVX group in negative mode (E) TIC of E2 group in positive mode (F) TIC of E2 group in negative mode. Figure S5. Score Scatter Plots for PCA-X analysis in (A) negative mode and (B) positive mode.
