# Supplementary material for: Metabolomics Coupled with Pathway Analysis Provides Insights into Sarco-Osteoporosis Metabolic Alterations and Estrogen Therapeutic Effects in Mice
Source: Biomolecules. 2021 Dec 28;12(1):41. doi: 10.3390/biom12010041 (PMC8773875; doi:10.3390/biom12010041)
Supplement: Supplementary file 1 [file biomolecules-12-00041-s001.zip › biomolecules-1483319-supplementary.pdf]

Metabolomics coupled with pathway analysis provides insights into sarco-osteoporosis metabolic alterations and estrogen therapeutic effects in mice

Ziheng Wei[1¶], Fei Ge[2¶], Yanting Che[3¶], Si Wu[4,*], Xin Dong[2,*], Dianwen Song[1,*]

[1] Department of Orthopedics, Shanghai General Hospital, Shanghai Jiao Tong University School of Medicine, Shanghai 201620, China
[2] School of medicine, Shanghai University, Shanghai 200444, China
[3] College of Sciences, Shanghai University, Shanghai 200444, China
[4] Department of Genetics, Stanford University School of Medicine, Stanford, CA, USA

[*] Corresponding authors:
siwu@stanford.edu (S.W), dongxinsmmu@126.com (X.D) and dianwen_song@126.com (D.S)
[¶] Ziheng Wei, Fei Ge and Yanting Che contributed equally to this work.

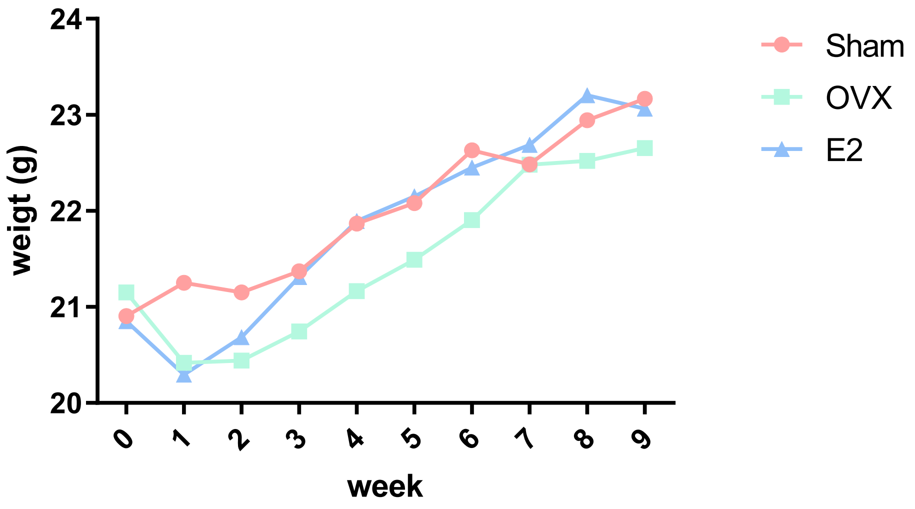

Figure S1. Body weight of mice in Sham, OVX and E2 group was recorded (n=6). No significant differences were observed (P＞0.1).

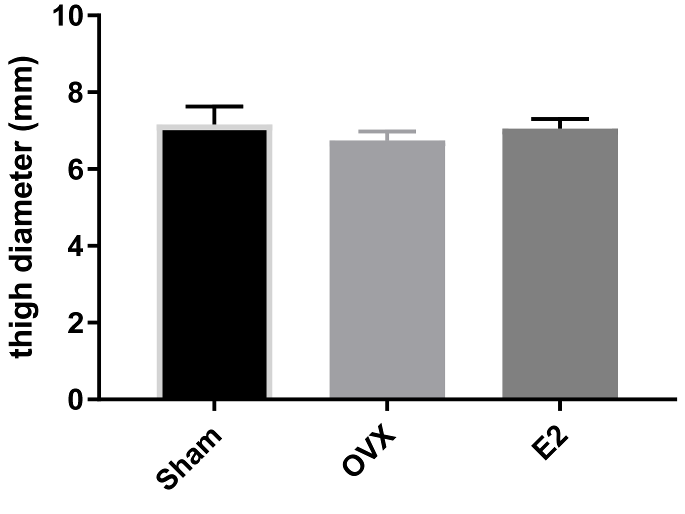

Figure S2. Thigh diameter of mice in Sham, OVX and E2 group was measured (n=6). No significant differences were observed (P＞0.05).

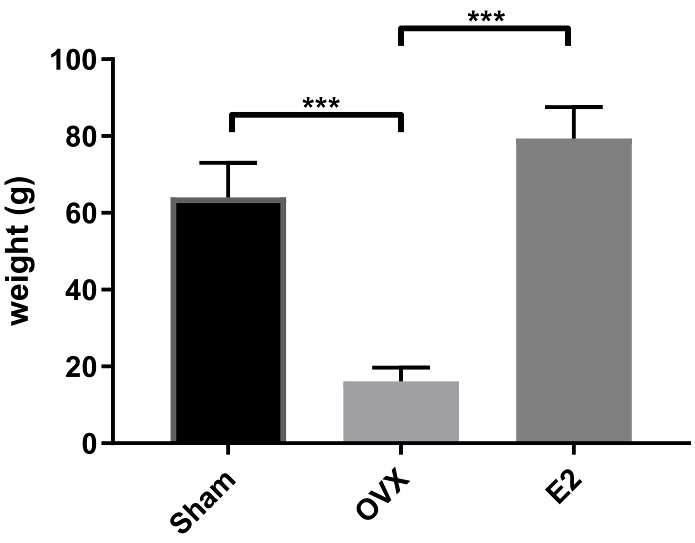

Figure S3. The average uterus weight of mice in Sham, OVX and E2 group was measured (n=6, P＜0.05).

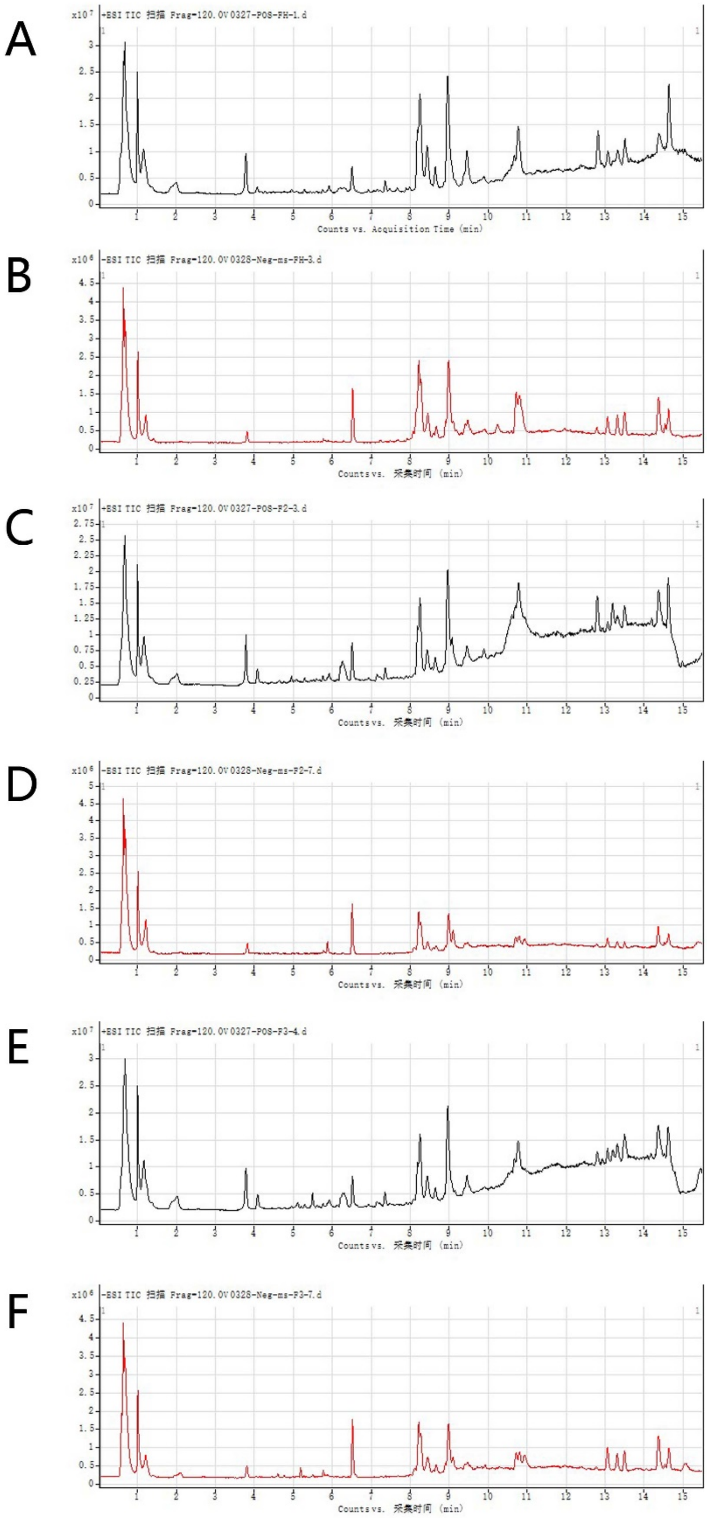

Figure S4. Representative total ion chromatograms of UPLC-Q-TOF/MS (A) TIC of Sham group in positive mode (B) TIC of Sham group in negative mode (C) TIC of OVX group in positive mode (D) TIC of OVX group in negative mode (E) TIC of E2 group in positive mode (F) TIC of E2 group in negative mode.

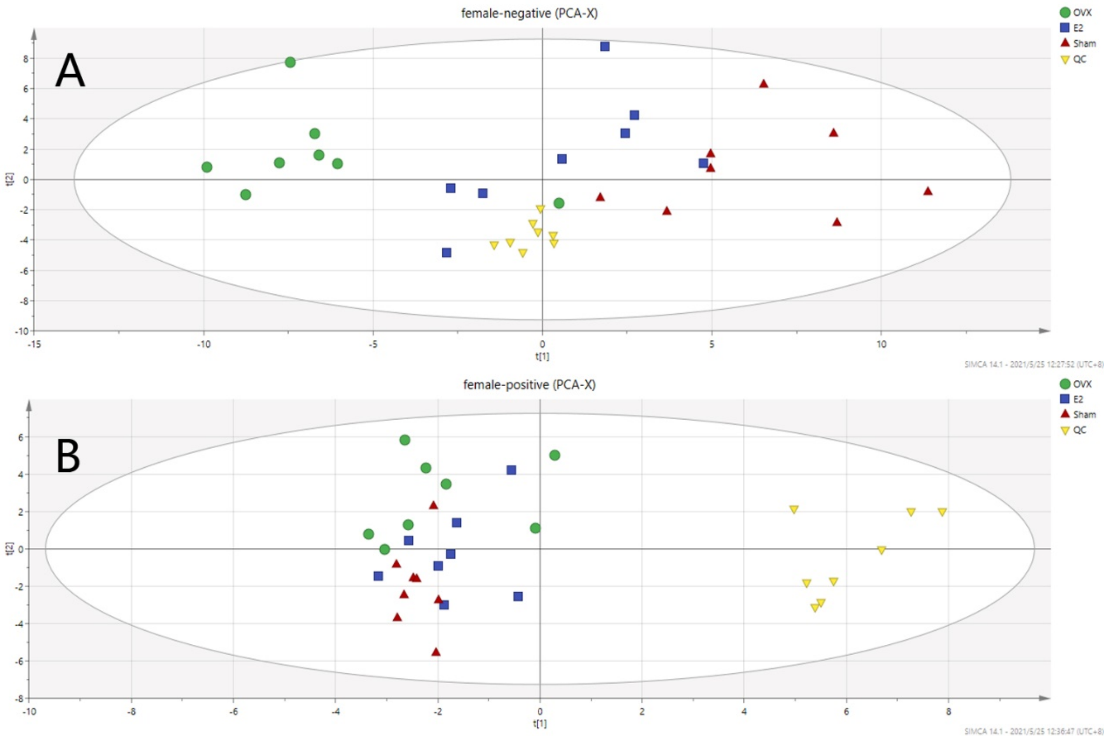

Figure S5. Score Scatter Plots for PCA-X analysis in (A) negative mode and (B) positive mode.
